# Noninvasive prediction of Blood Lactate through a machine learning-based approach

**DOI:** 10.1038/s41598-019-38698-1

**Published:** 2019-02-18

**Authors:** Shu-Chun Huang, Richard Casaburi, Ming-Feng Liao, Kuo-Cheng Liu, Yu-Jen Chen, Tieh-Cheng Fu, Hong-Ren Su

**Affiliations:** 1 Department of Physical Medicine and Rehabilitation, Chang Gung Memorial Hospital, Linkuo, Taiwan; 2 Rehabilitation Clinical Trials Center, Los Angeles Biomedical Research Institute at Harbor-UCLA Medical Center, Torrance, California USA; 3 Department of Neurology, Chang Gung Memorial Hospital and College of Medicine, Chang Gung University, Linkuo, Taiwan; 4 Department of Physical Medicine and Rehabilitation, Chang Gung Memorial Hospital, Keelung, Taiwan; 5 Institute of Statistical Science, Academia Sinica, Taipei, Taiwan; 6 Healthy Aging Research Center, Chang Gung University, Taoyuan, Taiwan

## Abstract

We hypothesized that blood lactate concentration([Lac]_blood_) is a function of cardiopulmonary variables, exercise intensity and some anthropometric elements during aerobic exercise. This investigation aimed to establish a mathematical model to estimate [Lac]_blood_ noninvasively during constant work rate (CWR) exercise of various intensities. 31 healthy participants were recruited and each underwent 4 cardiopulmonary exercise tests: one incremental and three CWR tests (low: 35% of peak work rate for 15 min, moderate: 60% 10 min and high: 90% 4 min). At the end of each CWR test, venous blood was sampled to determine [Lac]_blood_. 31 trios of CWR tests were employed to construct the mathematical model, which utilized exponential regression combined with Taylor expansion. Good fitting was achieved when the conditions of low and moderate intensity were put in one model; high-intensity in another. Standard deviation of fitting error in the former condition is 0.52; in the latter is 1.82 mmol/liter. Weighting analysis demonstrated that, besides heart rate, respiratory variables are required in the estimation of [Lac]_blood_ in the model of low/moderate intensity. In conclusion, by measuring noninvasive cardio-respiratory parameters, [Lac]_blood_ during CWR exercise can be determined with good accuracy. This should have application in endurance training and future exercise industry.

## Introduction

Blood lactate during aerobic exercise is the result of glycolytic metabolism, an anaerobic energy production pathway in muscle cells. Its concentration in muscle and blood reflect the extent of involvement of anaerobic metabolism. Blood lactate levels are also an important concern in exercise training.

Aerobic exercise intensity has been divided into three zones by ventilatory threshold 1(VT1) and ventilatory threshold 2(VT2) identified by breath-by-breath gas exchange measurement during incremental exercise testing^1^. In the moderate zone (between VT1 and VT2), the [Lac]_blood_ is increased but production and elimination rates reach equilibrium. VT2 is the highest possible intensity to achieve a steady [Lac]_blood_, termed maximal lactate steady state^2–4^. Studies demonstrated that the intensity of endurance training between VT1 and VT2 significantly improved fitness among the untrained subjects^5,6^. In addition, in the high zone above VT2, the [Lac]_blood_ accumulates rapidly and fatigue is forthcoming. VT1(also lactate threshold or anaerobic threshold) and VT2(also respiratory compensation point or onset of blood lactate accumulation) correspond to 1~2 and 4 mM/liter blood lactate concentration respectively^2^. Accordingly, it is valuable to noninvasively obtain the numerical value of [Lac]_blood_ during endurance training.

The current investigation attempted to establish a novel mathematical model to estimate [Lac]_blood_ noninvasively. During exercise, an extremely complicated relationship exists between [Lac]_blood_ and tidal volume (V_T_), breathing frequency (BF), exercising heart rate(ExHR), resting HR(ReHR), and anthropometric characteristics such as body weight. Exercise intensity plays a key role in delineating the complex interaction among these physiologic variables. As intensity increases, HR, Bf, V_T_ and [Lac]_blood_ all increase but with different trajectories. Accordingly, we hypothesized that [Lac]_blood_ is a function of cardiopulmonary variables, exercise intensity and anthropometric characteristics.

## Methods

Thirty-one healthy male and female participants between 20 and 50 years-old were recruited by convenience sampling (Table 1). Those with cardiovascular illness were excluded. The experiment protocol was approved by the Chang Gung Memorial Hospital Institutional Review Board. All the subjects provided written informed consent after receiving an oral and printed explanation of the experimental procedures. This research was performed in accordance with the ethical standards of the Declaration of Helsinki.

**Table 1:** Anthropometric data.

age	(year)	33 ± 9
gender	(male: female)	14:17
body height	(centimeter)	165 ± 9
body weight	(kilogram)	62.7 ± 11.6
BMI	(kg/m^2^)	22.8 ± 2.7

Mean ± standard deviation

### Cardiopulmonary Exercise test and blood lactate measurement

Every participant underwent four cardiopulmonary exercise tests on a cycle ergometer (Ergoselect 150 P, Germany) on different days: one incremental and three constant work rate (CWR) tests. CWR tests were of mild, moderate and high intensity. Each subject was instructed to refrain from exercise for 12 hours before each test. The incremental exercise test comprised 1 minute of unloaded pedaling followed by an incremental increase in work rate of 15 watts per minute until exhaustion; thereby the peak work rate was determined. The VO_2peak_ was defined by the following criteria: (i) VO_2_ increased by less than 2 mL/kg/min over at least 2 min, (ii) HR exceeded 85% of its predicted maximum, (iii) the respiratory exchange ratio exceeded 1.15, or (iv) some other symptom/sign limitations^7^. Subsequently, each subject performed three CWR exercise tests: 15-minute low-constant load at 35% peak work rate, 10-minute moderate-constant load at 60% peak work rate and 4-minute high constant-load at 90% peak work rate. The CWR intensity of low (35%), moderate (60%) and high (90%) were chosen based on three zones: below VT1, between VT1 and VT2, and above VT2^1,2^. In the majority of healthy people, VT occurs at 40–60% of VO_2max_^8^. RCP has been reported to be 61.3 to 85.4% of VO_2max_ in healthy subjects^3,9^. Minute ventilation (*V*_E_), oxygen uptake ($\dot{{\rm{V}}}{{\rm{O}}}_{2}$), and carbon dioxide production ($\dot{{\rm{V}}}{{\rm{C}}{\rm{O}}}_{2}$) were measured breath-by-breath using a computer-based system (MasterScreen CPX, Cardinal-health Germany). Heart rate (HR) was determined from the R-R interval of a 12-lead electrocardiogram (CardioSoft, GE, Wilwaukee, USA). Arterial blood pressure was measured every two minutes using an automatic blood pressure system (Tango, SunTech Medical, UK), and arterial O_2_ saturation was monitored continuously by a finger pulse oximetry (model 9500, Nonin Onyx, Plymouth, Minnesota). End-exercise values were determined as the average of the final 30 seconds of exercise. In the CWR tests, venous blood was sampled mostly from an antecubital vein or in a few cases from a dorsal interosseous metacarpal vein for [Lac]_blood_ assay 30–60 seconds after the end of constant work rate exercise tests. The sample was collected in NaF/K_3_EDTA tubes and then placed on ice. The whole blood was centrifuged within 90 minutes to obtain plasma, which was stored at 4 °C. [Lac]_blood_ was measured by the enzymatic method within 14 days after sampling (DXC880i).

### Mathematical model for lactate estimation

We propose a novel model to estimate [Lac]_blood_ by noninvasively-measured physiologic signals including V_T_, BF, ReHR, ExHR, age, body mass index (BMI) and sex at the end of the CWR testing. The model is based on an exponential regression method combined with Taylor expansion^10^ to find the best predictors of [Lac]_blood_.

#### Exponential function and Taylor expansion

An exponential regression model between [Lac]_blood_ and the physiologic signals at CWR testing was examined, including BF, VT, BMI, age, ReHR and ExHR. We asserted that the connection between [Lac]_blood_ and various physiologic signals can be formulated into a poly-exponential function^11,12^ and defined by equation 1.

1 $$f(x)={e}^{-Ax}$$

where *x* is a matrix of the independent variables, *A* is the weighting matrix corresponding to each independent variable, and *f*(*x*) is [Lac]_blood_. A supervising gradient descent was adopted to equation 1 to solve the model *A* with a full-rank matrix by x and *f*(*x*)^13^. In order to solve the equation 1 efficiently, Taylor expansion was applied to transfer equation 1 into polynomial form for linear solution. Taylor expansion can be expressed in equation 2 for x = a.

2 $$f(x)=f({\rm{a}})+\frac{f^{\prime} (a)}{1!}(x-a)+\frac{f^{\prime\prime} (a)}{2!}{(x-a)}^{2}+\frac{f^{\prime\prime} \,^{\prime} (a)}{3!}{(x-a)}^{3}+\ldots $$

Equation 1 can be transformed into equation 3 for cubic approximation polynomials by Taylor expansion.

3 $$Y=f(x) \sim {a}_{0}-{a}_{1}x+{a}_{2}\frac{{x}^{2}}{2!}-{a}_{3}\frac{{x}^{3}}{3!}$$

Equation 3 is rewritten in matrix form as follows^14^.

4 $${\rm{Y}}={\rm{AX}}$$

where $${\rm{A}}=[{a}_{0}-{a}_{1}\,\frac{{a}_{2}}{2!}\,\frac{-{a}_{3}}{3!}]$$ and $${\rm{X}}={[1x{x}^{2}{x}^{3}]}^{T}$$.

#### Linear regression

Equation 4 is the relationship between the multiple independent variables X and [Lac]_blood_; the model A can be solved by linear regression analysis^15^ Additionally, leave-one-out cross validation was applied to avoid overfitting8–10 as follows:

5 $$A={({X}^{T}X)}^{-1}{X}^{T}Y$$

In the training step, physiologic, anthropometric variables and [Lac]_blood_ from the database involving CWR testing at low, moderate and high intensity were employed to construct the A matrix and solve for regression coefficients. In the testing step, estimated blood lactate concentration ([Lac]_estimate_), $\hat{Y}=AX$, was used to verify the difference between true and the estimated lactate value by error and variance.

The error distance d is

6 $${\rm{d}}={\rm{Y}}-\hat{Y}$$

and the sum of squares D of all data is

7 $$D={\sum \Vert {y}_{i}-A{x}_{i}\Vert }^{2}$$

8 $${\sigma }^{2}=\frac{D}{n-1}$$

Where *σ*^2^ is the variance. The solution matrix A in equation 5 is satisfied with the minimum D in order to get the minimum error variance.

#### Leave-one-out cross validation

Additionally, leave-one-out cross validation was applied to avoid overfitting^16–18^, which is briefly described as follows:

- If k observations are recorded, one is used for testing and the other k-1 observations are for training.
- The above procedure is repeated k times in the k observations for testing and training.
- Mean square error is used for justification in order to get the best model. The mean square error of the leave-one-out cross validation models was determined at about 0.27 in the low & moderate intensity model and 0.25 in the high intensity model.

#### Weighting factor

In the linear regression, the selected 8 independent variables were used to build the model for lactate estimation. The 8 weightings directly acquired from the model A in equation 5 corresponding to the 8 factors are the absolute positive or negative weighting. Additionally, normalized weighting was computed, which was defined by each absolute positive or negative weighting divided by sum of all the absolute positive or negative weighting. These two kinds of weighting were applied to quantify the impact of the 8 independent variables in lactate estimation.

#### Implementation

Modeling was performed in MATLAB 2014b (MathWorks®, Natick, Massachusetts, United States).

### Statistics

Linear correlation and Bland-Altman plots^19^ were employed to show the validity of estimated blood lactate level. Descriptive statistics were also used. Data are presented as mean ± standard deviation.

## Results

The average work rate corresponding to low, moderate and high CWR was 66 ± 29, 107 ± 46 and 146 ± 66 watts. The mean [Lac]_blood_ was 3.7 ± 2.3, 6.9 ± 4.2 and 10.4 ± 4.1 mM/liter for the three intensities (Table 2).

**Table 2:** Physiologic variables during graded and constant-work rate exercise test.

**Incremental**		
Maximal VO_2_	(ml/min/kg)	30.1 ± 11.9
Maximal work rate	(watt)	187 ± 78
VO_2_ at lactate threshold	(ml/min/kg)	21.9 ± 6.7
Work rate at lactate threshold	(watt)	119 ± 53
[Lac]_blood_ at lactate threshold	mM/liter	3.0 ± 1.0
**CWR**		
Work rate at LC	(watt)	66 ± 29
Work rate at MC	(watt)	107 ± 46
Work rate at HC	(watt)	146 ± 66
blood lactate concentration at LC	(mM/liter)	3.7 ± 2.3
blood lactate concentration at MC	(mM/liter)	6.9 ± 4.2
blood lactate concentration at HC	(mM/liter)	10.4 ± 4.1

Mean ± standard deviation

CWR: constant work rate

LC: low-constant exercise test 35% maximal work rate 15′.

MC: moderate-constant exercise test 60% maximal work rate 10′.

HC: high-constant exercise test 90% maximal work rate 4′.

**Figure 1:**
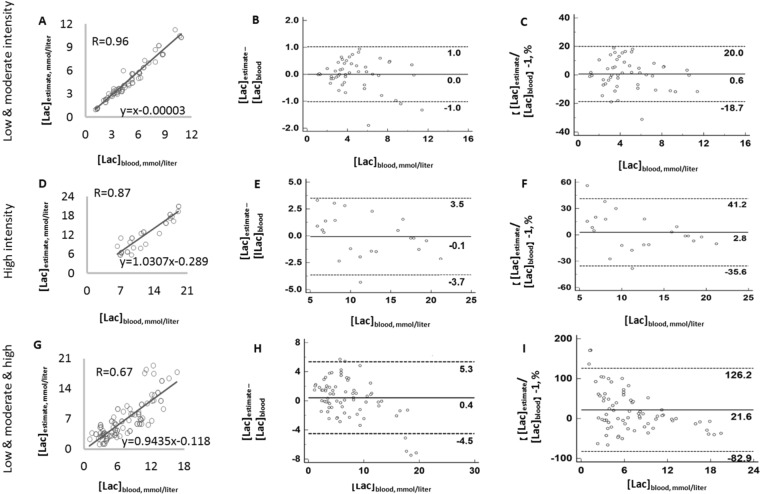
Model estimates of blood lactate concentration compared with the measured value. (**A**–**C**) are low combined with moderate intensity CWR tests. (**D**–**F**) are those for high-intensity exercise. (**G**–**I**) is the model obtained when the data of three different intensities are combined. Scatter plot are demonstrated in (**A**,**D**,**G**). Agreement using Bland-Altman plot are shown in (**B**,**C**,**E**,**F**,**H**) and (**B**,**E**,**H**,**I)** show the difference between estimated and measured value. (**C**,**F**,**I**) are the percentage of estimation error. The dark solid horizontal lines in each Bland-Altman plot represent average bias whereas the dotted lines stand for average bias ± 1.96 standard deviation (95% upper and lower limit). The standard deviation in (**B**,**E**,**H**) are 0.52, 1.83 and 2.51 mmol/liter. The standard deviation in (**C**,**F**,**I**) are 0.1, 0.2 and 0.53.

The mathematical analysis showed that, if low, moderate and high intensity are processed together, the fitting error was large (Fig. 1G–I). However, if we fit the data in low- and moderate- intensity CWR (Fig. 1A–C) together, and fit the high-intensity data (Fig. 1D–F) separately, the fitting error becomes much smaller. In these two conditions, both models fit the data quite well, especially low and moderate intensity, in which the standard deviation of fitting error is 0.52 mmol/liter (Fig. 1B). This indicates that a very different relationship exists between [Lac]_blood_ and these measured variables under the two conditions: low/moderate, and high intensity.

**Figure 2:**
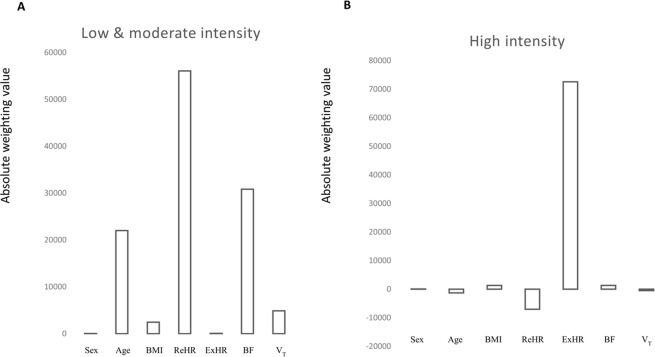
Absolute positive or negative weighting of each variable in determining blood level of lactate. BMI: body mass index; ExHR: exercising heart rate; BF: breathing frequency; V_T_: tidal volume

Figure 2A presents the absolute weighting of each variable in determining [Lac]_blood_. In low & moderate constant-load intensity, ReHR, BF and age have the greatest positive influence. On the other hand, during high-intensity condition, ExHR alone has a significant impact (Fig. 2B).

## Discussion

In the present investigation, responses to 31 trios of CWR exercise tests in 31 subjects were employed to construct a mathematical model to estimate [Lac]_blood_ from noninvasive measurement. The database is comprised of low, moderate and high intensity exercise tests. The model is based on exponential regression method combined with Taylor expansion. The independent variables included ExHR, ReHR, Bf, VT, BMI, age and sex. Excellent fitting was achieved in the conditions of low and moderate intensity in one model, and high-intensity in another model. The standard deviations of fitting error in the former condition is 0.52 mmol/liter; and is 1.82 in the latter condition, which are acceptable for the purposes of specifying exercise training targets.

This result implies that exercise intensity is a significant determinant in the complex relationship between [Lac]_blood_ and cardiopulmonary variables. Poor fitting was obtained when we attempted to construct a single mathematical model including of three exercise intensities. Three intensity zones divided by VT1 and VT2 has been reported to have distinct differences in sympathetic stress load, motor unit involvement, and duration to fatigue^2^. The difference could be too large to be mathematically processed in a single model. Additionally, regarding to the model construction, the accuracy of [Lac]_blood_ was also affected by the amount and consistency of the training data. The database in the present study is small compared to the sample size commonly employed in deep learning. The consistency of our data, as revealed by the leave-one-out validation, is good. Therefore, increase of the sample size will improve the precision of the estimation, especially in the high-intensity database. It is also worth mentioning that multiple linear regression was attempted at first. Little or low correlation was found between almost all the independent variables versus [Lac]_blood_ (the highest correlation coefficient is 0.54)_._ Accordingly, a more complicated mathematical method is adopted in the analysis.

Modeling weight distribution analysis showed that BF is indispensible and even more important than heart rate as an independent variable in the conditions of low/moderate intensity. The advancement of wearing device is progressing rapidly, exercising BF may be acquired conveniently soon though we are unaware of any wearable devices that measures currently. The mathematical model is constructed with a view to applying it in the exercise industry. Therefore, $\dot{{\rm{V}}}{{\rm{O}}}_{2}$ and $\dot{{\rm{V}}}{{\rm{C}}{\rm{O}}}_{2}$ are not included because they are quite impossible to obtain without a gas analysis system.

The algorithm of the current study could be applied in the threshold training model^2^ of cycling endurance training. The suitable intensity is to keep [Lac]_blood_ in the range of VT1 and VT2, especially for the untrained people. The corresponding [Lac]_blood_ are 1~2 and 4 mM/liter^2^. SD of fitting error in the low/moderate model is 0.52 mM/liter. Considering the width of the middle zone, the fitting error should be acceptable. Increase the sample size will further minimize the fitting error. Another possible application could be in the CWR testing. Estimated [Lac]_blood_ may be used as a criterion to judge whether the subject approaches maximal effort when gas analysis to measure $\dot{{\rm{V}}}{{\rm{O}}}_{2}$ plateau is not available. A variety of [Lac]_blood_ cut-off values have been proposed. Most of the criteria are around 8 mM/liter^20,21^. Further study is needed to prove this idea.

The mathematical methodology employed in this study should apply to other CWR conditions during exercise. The most common scenario is in treadmill exercise in which speed and slope are fixed. Further, some steppers are provided with constant-power modes. Additionally, wearable device that measure physiological responses are being developed. Our model to estimate [Lac]_blood_ would be relevant to a free ambulation constant work rate task in which cardiac and respiratory responses are measured.

There are several study limitations. First, the sample size is relatively small. However, good validity is still attainable, which suggests that the mathematical model employed in the present study works. A large database should be acquired to increase the accuracy, especially in the model of high intensity. Secondly, equation (regression coefficient or A value) obtained in this study may only be relevant to CWR cycle ergometer exercise. Studies should be undertaken to test its validity during other exercise modalities (e.g., treadmill, walking). Third, prospective validation procedures were not performed in this study. Nonetheless, if we do prospective validation, those data can be pulled into the learning model to generate a new regression coefficient. Leave-one-out cross validation^8^ was already applied to our model to determine the overfitting statistical learning.

## Conclusion

This is the first study to establish a mathematical model in predicting the numerical values of [Lac]_blood_ during exercise. By measuring noninvasive cardio-respiratory parameters and including some anthropometric factors, [Lac]_blood_ during constant work rate exercise can be determined with good validity by exponential regression combined with Taylor expansion. These experimental finding should have application in designing intensities during endurance training and future exercise industry.

## Supplementary information

Supplementary Info File #1

## Data Availability

The datasets generated during and/or analysed during the current study are not publicly available due to its potential commercial interest but are available from the corresponding author on reasonable request.
