# Supplementary material for: Noninvasive prediction of Blood Lactate through a machine learning-based approach
Source: Sci Rep. 2019 Feb 18;9:2180. doi: 10.1038/s41598-019-38698-1 (PMC6379358; doi:10.1038/s41598-019-38698-1)
Supplement: Supplementary file 1 — Supplementary Info File #1 [file 41598_2019_38698_MOESM1_ESM.pdf]

Supplementary material

Noninvasive prediction of Blood Lactate through a machine learning-based approach

Shu-Chun Huang[a,f], Richard Casaburi[b], Ming-Feng Liao[c], Kuo-Cheng Liu[a], Yu-Jen Chen[a], Tieh-Cheng Fu[d,f] and Hong-Ren Su[e *]

[a] Department of Physical Medicine and Rehabilitation, Chang Gung Memorial Hospital, Linkuo, Taiwan

[b] Rehabilitation Clinical Trials Center, Los Angeles Biomedical Research Institute at Harbor-UCLA Medical Center, Torrance, California, USA

[c] Department of Neurology, Chang Gung Memorial Hospital and College of Medicine, Chang Gung University, Linkuo, Taiwan

[d] Department of Physical Medicine and Rehabilitation, Chang Gung Memorial Hospital, Keelung, Taiwan

[e] Institute of Statistical Science, Academia Sinica, Taipei, Taiwan

[f] Healthy Aging Research Center, Chang Gung University, Taoyuan, Taiwan

*** Correspondence author:** Professor, Hong-Ren Su, Institute of Statistical Science,

Academia Sinica, Taipei, Taiwan. 128 Academia Road, Section 2, Nankang, Taipei 11529, Taiwan. TEL # 886-2-29946697. E-mail address: suhongren@gmail.com

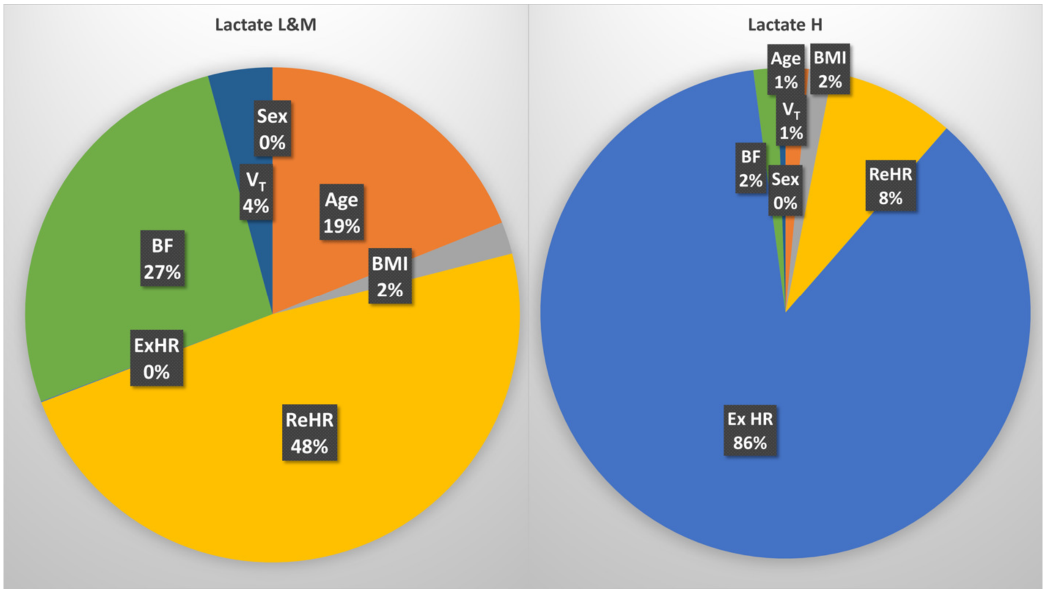

Influence of each variable employed in the $[Lac]_{blood}$ estimation is normalized and demonstrated in the pie chart in which L&M is for the conditions of low and moderate intensity exercise whereas H is for high-intensity exercise.
